# Environmental Impact of DEET: Monitoring in Aquatic Ecosystems and Ecotoxicity Assessment

**DOI:** 10.1021/acsestwater.5c00489

**Published:** 2025-10-03

**Authors:** Tereza Motúzová, Anna Gavlová, Kateřina Smutná, Lucie Řepecká, Martina Vráblová

**Affiliations:** † Institute of Environmental Technology, CEET, VSB-Technical University of Ostrava, 17. listopadu 15/2172, Ostrava-Poruba, 708 00, Czech Republic; ‡ Faculty of Materials Science and Technology, 48278 VSB-Technical University of Ostrava, 17. listopadu 15/2172, Ostrava 70800, Czech Republic

**Keywords:** DEET, HPLC-MS/MS, water quality monitoring, WWTP, ecotoxicity

## Abstract

Pollution of surface watercourses and reservoirs with pesticides is a serious global problem. *N*,*N*-Diethyl-*meta*-toluamide (DEET), a widely used repellent against mosquitoes and ticks, can enter aquatic ecosystems from point sources when used outdoors but especially from wastewater from laundry and personal hygiene. This research was focused on the monitoring of DEET in surface water, sediments, plants growing on the banks, gray water and in a wastewater treatment plant (WWTP), both in water and sewage sludge. For identification and quantification of DEET, liquid chromatography coupled with mass spectrometry (HPLC-MS/MS) was used. The study was complemented by determining DEET ecotoxicity to nontarget organisms (*Vibrio fischeri*, *Sinapis alba*, and *Eisenia andrei*). The research has demonstrated the presence of DEET in all investigated areas in water in a concentration range of up to 32.18 μg L^–1^. While the concentrations of DEET found do not possess acute toxic effects, it is imperative to acknowledge its potential for chronic effects, toxicity of any possible degradation products, and synergistic effects with other pollutants present in the environment, especially in the aquatic ecosystem.

## Graphical abstract

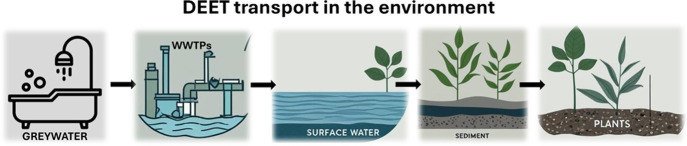

## 1 Environmental Implication

*N*,*N*-Diethyl-*meta*-toluamide (DEET) is a widely used tick and mosquito repellent, which results in its release into the environment, where it may pose a potential risk to nontarget organisms. This research focused on monitoring DEET in various environmental matrices – surface water graywater, wastewater, sediments, sludge, and plants. Ecotoxicity studies have shown possible chronic effects on nontarget organisms. We hope that this manuscript will be suitable for publication in your journal, as it is a hazardous chemical, especially for nontarget organisms, with long-term persistence in the environment.

## 2 Introduction

DEET (*N*,*N*-diethyl-*meta*-toluamide, CAS No. 134–62–3) was developed by the US military in 1946 and has been commercially available as an effective repellent since 1957. It is used to repel mosquitoes, ticks and other insects, thereby protecting against the transmission of diseases such as malaria, dengue or West Nile fever.^1–4^ The mechanism of action consists of disrupting the insect’s ability to detect host odors.^1,4^ In commercial products, DEET is available in various ranges of concentrations from 5% to 100%, with higher concentrations increasing the duration of protection but not its effectiveness,^5^ whereby products with DEET concentration below 40% being the most commonly used. Formulations containing DEET are applied to either the skin or clothing. DEET is mostly used to protect humans, but occasionally it is also used on animals, such as pets and livestock, in order to protect them from mosquito bites, suggesting another source of contamination is detected in rural areas.^6^

Due to the extensive use of DEET in households and industry, this substance enters the environment mainly through wastewater and surface washes after the application of repellents.^7,8^ Detection of DEET has been confirmed in rivers, lakes, and even wastewater treatment plant effluents, indicating its persistent nature and ability to overcome common water treatment processes.^8–10^ DEET is widely used all over the world, which is why it is often detected in the aquatic environment in the concentration range of ng L^–1^ to μg L^–1^. More than 80% of DEET is excreted into the environment after application.^11^ Approximately 79% of DEET remains in surface water and 21% in soil; it is only marginally present in the air and breaks down quickly. The degradation half-life (DT_50_) typically ranges from a few days to weeks depending on conditions, meaning that DEET is not long-term persistent in nature.^12^ The biological degradation of DEET involves various mechanisms such as *N*-oxidation, *N*-dealkylation, and demethylation on the benzene ring. Available data indicate that the main metabolites formed (primarily 3-methylbenzoate) are relatively well degradable and accumulate only transiently in the environment.^4,13^

DEET has relatively low acute toxicity to aquatic organisms, but its presence in the aquatic environment can cause sublethal effects, such as disruption of reproduction and behavior of aquatic animals.^2,4^ The presence of DEET can disrupt ecosystems and contribute to long-term exposure of nontarget organisms, raising concerns about its accumulation in the environment.^10^ DEET has also potential carcinogenic properties for humans and it can cause confusion, headache, convulsions, tremors, disorientation, as well as skin problems such as hives.^14^ After exposure to the cells of the nasal mucosa in children, coma and seizures can occur even at a low dose.^15^ For this reason, the concentration of DEET in the preparations is regulated. People aged 2–12 years are only allowed to use products with a DEET concentration of up to 10%, products with a DEET content of 30% can only be used by people over 12 years old.^16^

DEET is commonly found in aquatic environments worldwide, including drinking water, streams, and seawater.^6^ Its presence raises concerns about its ecotoxicity and potential risks to nontarget organisms. Ecotoxicological effects have been studied in zooplankton and crustaceans (e.g., *Daphnia magna*, *Macrobrachium nipponense*), amphibians and fish (e.g., *Rhodeus sinensis*), invertebrates (e.g., *Chironomus riparius*, *Limnodrilus hoffmeisteri*), and algae, such as *Pseudokirchneriella subcapitata*, a freshwater alga commonly used in ecotoxicological tests, and *Chlorella protothecoides*, a unicellular green alga utilized in biotechnology and ecotoxicological studies.^3,12^ The study by Gao et al.^3^ indicates that the most sensitive organisms to DEET are algae > crustaceans > amphibians > fish > insects > annelids. However, for some organisms, including those in our study, sufficient data is still lacking. The ecotoxicity of DEET for *Eisenia andrei* has not yet been thoroughly studied, although DEET can leach into the soil and threaten its biota. For this organism, ecotoxicological effects have been investigated only in relation to NSAID-type substances, where varying levels of toxicity were observed in earthworms. Similarly, for the bioluminescent bacterium *Vibrio fischeri*, which serves as a model organism for studying ecotoxic effects on bacteria, research has mainly focused on DEET in the context of its photocatalytic degradation using TiO_2_, as observed in the study by Medana et al.^17^ This experiment demonstrated that DEET degradation products exhibited ecotoxic effects, emphasizing the importance of considering not only the parent compound but also its transformation products in environmental risk assessments. The study of DEET ecotoxicity on terrestrial plants, specifically white mustard (*Sinapis alba*), a widely used organism in ecotoxicological studies, remains very limited. Initial research suggests a negative impact of DEET on seed germination and plant growth, but available data are still insufficient, and further research is necessary.^18^ Overall, there is a lack of comprehensive studies assessing the ecotoxic effects of DEET on *Sinapis alba*, *Eisenia andrei*, and *Vibrio fischeri*. To mitigate the environmental impact of DEET, enhanced wastewater treatment technologies are essential.^6,19^

Removing DEET from wastewater is a challenging task because common treatment technologies such as biological oxidation or sedimentation are not effective enough.^2,9^ Studies show that advanced methods such as ozonation, UV radiation, photocatalysis, and the use of activated carbon are more effective, but more expensive.^2,4,20^ For example, the sorption of DEET onto activated carbon allows the concentration of DEET to be reduced from 100 μg L^–1^ to less than 1 ng L^–1^, which may be suitable for drinking water treatment.^2^

DEET concentrations in wastewater can fluctuate seasonally. During the summer, DEET use accounts for nearly 60% of all use during the year, while during the winter months, DEET use accounts for <5%.^4,5^ In sewage sludge, DEET can be present in concentrations from 0.1 to 1.5 μg g^–1^ dry matter.^2^

This research aimed to monitor the occurrence of repellent pesticide DEET in water bodies (surface, wastewater, and gray water), sediments, sewage sludge, and bank-inhabiting plants. For this purpose, the goal was to create and validate analytical methods for determining the DEET concentration in different matrices during the annual season. In addition, the ecotoxic effects of DEET were tested to assess the environmental effects of its occurrence.

## 3 Materials and Methods

### 3.1 Chemicals and Materials

Acetonitrile for LC-MS (purity 99.95%) was purchased from Chromservis (Czech Republic), methanol for LC-MS (purity 99.95%) was purchased from Biosolve (Netherlands), and ammonium formate for LC-MS (purity 98 – 100%), DEET (purity ≥ 95%) were purchased from Sigma-Aldrich (USA). MgSO_4_ (purity ≥ 99%), C_6_H_6_Na_2_O_7_·1.5 H_2_O (purity ≥ 99%), and NaCl (purity ≥ 99.5%) were purchased from Carl ROTH (Germany) and C_6_H_6_Na_3_O_7_·2H_2_O (purity 99%) was purchased from Penta (Czech Republic). DEET (purity 97%, Thermo Scientific, MA, United States) and ethanol (96% vol, VWR International S.A.S., France) were used for ecotoxicity tests.

### 3.2 Monitoring of DEET in Surface Water

Monitoring of DEET in surface waters was carried out monthly over a period of 14 months, encompassing seven collection sites situated within the Moravian-Silesian and Olomouc regions of the Czech Republic. The GPS coordinates of the individual collection sites are given in Table 1. Surface water samples were obtained from rivers, small watercourses, and ponds. One water sample was collected from each point from a depth of 10–15 cm below the surface. Samples were collected in glass containers made of silicate glass with a metal lid. After rinsing the container several times, approximately 500 mL of sample was collected, which was then left in the dark and cold (4 °C) until further analysis.

**Table 1:** Retention Times of Both MRM Transitions and LOQ and LOD for Water, Sediment, and Plant Samples

sampling point	experimental site (city – name of the body of water)	GPS coordinates
point 1	Bohumín - Odra (river)	49.948417, 18.333158
point 2	Bohumín - Záblatí (pond)	49.880949, 18.373840
point 3	Bohuslavice - Bohuslavický potok (stream)	49.944131, 18.142446
point 4	Opava - Kateřinský potok (stream)	49.949389, 17.931167
point 5	Ostrava - Opava (river)	49.839465, 18.198504
point 6	Potštát - Harta (pond)	49.634665, 17.638398
point 7	Potštát - Rybník u hřiště (pond)	49.640628, 17.646015

The water samples were stored in a refrigerator at a temperature of 4 °C until further preparation. On the day of the analysis, the samples were filtered using a vacuum filtration apparatus (Scharlau, Spain) with glass microfiber filters (mesh of 1.2 μm; filtraTECH, France) to remove any solid particles; 100 mL of filtrate was taken for further preanalytical steps.

#### 3.2.1 Solid-Phase Extraction

Solid-phase extraction (SPE) is a powerful technique for the targeted isolation of an analyte from a sample and its subsequent concentration with a small sample volume being sufficient for analysis. Extraction columns are formed by a sorbent on which the given analyte is captured due to its physical and chemical properties. Sorbents are chosen according to the properties of the analyte.^21^

Subsequently, solid phase extraction was performed using EnvirElut Pesticides SPE extraction columns (Agilent, USA), which were placed in a vacuum manifold (Chromabond, Germany), with an attached vacuum pump (MEVACS M46, MEDIST, Slovakia).

The extraction was carried out in several steps. First, conditioning of SPE columns was performed with 2 mL of MeOH and 2 mL of H_2_O. Then, 100 mL of water sample was applied to the column with the addition of 25 μL of an internal pesticide standard of *c* = 1 μg mL^–1^. A flow rate of approximately 5 drops per minute was used to ensure a sufficiently long sample retention time. Subsequently, a vacuum was applied for 10 min to dry the column. In the last step, elution was carried out using 2 mL of MeOH in plastic disposable tubes.

The solution was evaporated to dryness by using a stream of nitrogen in a sample concentrator (Stuart, Cole-Parmer, USA). The residue was reconstituted in 100 μL of ultrapure water and taken for analysis.

#### 3.2.2 LC-MS/MS Analysis

An HPLC gradient liquid chromatograph Nexera X2 series (Shimadzu, Japan) with a QTRAP 6500+ mass detector (Sciex, Canada) was used for the analysis. A Synergy Fusion RP 80Å (50 × 2 mm, 4 μm) analytical column (Phenomenex, USA) was used for the analysis alongside a binary mobile phase consisting of 5 mM ammonium formate in MeOH (MF A) and 5 mM ammonium formate in H_2_O (MF B). The applied gradient was as follows: MF B: 0 → 0.5:90% B; 0.5 → 1:60% B; 1 → 8:10% B; 8.2 → 9:90% B. The analysis was 9 min long, and the injection was 10 μL. For the mass-spectrometric detection multiple reaction monitoring (MRM) type of scan was used. Two MRM transitions were used for DEET analysis: one for quantification and one for qualification. Electrospray in positive mode was used for the ionization. The MS operational settings were as follows: capillary voltage: 5.5 kV, capillary temperature: 450 °C, the nebulizer gas pressure: 50 psi, and the heater gas pressure: 60 psi. The quantification was done using the inner standard (IS) method. A six-point calibration curve was assembled with the lowest concentration being 20 μg L^–1^ and the highest 200 μg L^–1^. The MRM transitions, retention time, and the calculated LOQ (using a standard deviation of the lowest point method) are listed in Table 2.

**Table 2:** Retention Times of Both MRM Transitions and LOQ and LOD for Gray Water and WWTP Samples

				water		sediment and plants	
analyte	Q1 (*m*/*z*)	Q3 (*m*/*z*)	retention time (min)	LOD (ng L^–1^)	LOQ (ng L^–1^)	LOD (μg kg^–1^)	LOQ (μg kg^–1^)
DEET 1	192.185	118.600	5.25	0.02	0.06	4	12
DEET 2	192.106	90.800					

### 3.3 Monitoring of DEET in Sediments

Monitoring of DEET in sediments was carried out three times per year at the same locations as those for surface water samples. Sediment samples were collected in glass sample boxes. Subsequently, they were dried to a constant weight at laboratory temperature. After being dried, they were sieved. The fraction below 2 mm was used for analysis. Ten g of dry matter was weighed for subsequent analysis. The samples were subsequently processed using a modified QuEChERS (Quick, Easy, Cheap, Effective, Rugged, and Safe) method.

#### 3.3.1 QuEChERS

QuEChERS represents an efficient sample preparation method that enables the isolation of an analyte from complex matrices. The method consists of two basic steps: (a) extraction step – separation using a salting-out reaction; (b) solid-phase dispersion extraction step, including a combination of sorbents and salts. The final step is centrifugation, filtration, and/or precipitation.^22^

Ten g portion of the dried material was placed in a polypropylene screw tube, into which 10 mL of water and 10 mL of acetonitrile were subsequently added. The mixture was then shaken on a rotary shaker (3500, VWR, USA). After shaking, 4 g of MgSO_4_, 0.5 g of C_6_H_6_Na_2_O_7_·1.5H_2_O, 1 g of NaCl, and 1 g of C_6_H_6_Na_3_O_7_·2H_2_O were added. The samples were then shaken for another 10 min and centrifuged for 50 min in a centrifuge (5810 R, Eppendorf, Germany). After centrifugation, the sample was divided into aqueous and organic phases.^22^

The aqueous phase was collected by using a dropper and subsequently processed by using SPE extraction.

#### 3.3.2 Solid-Phase Extraction

SPE extraction was performed in the same way as that for surface water samples. First, SPE columns were treated with 2 mL of MeOH and 2 mL of H_2_O. Subsequently, the collected aqueous phase was applied to the column with the addition of 25 μL of the internal pesticide standard *c* = 1 μg mL^–1^. The volume of the collected aqueous phase was approximately 3–5 mL. A flow rate of approximately 5 drops per minute was used to ensure a sufficiently long sample retention time. Subsequently, a 10 min vacuum was applied to dry the column. In the final step, elution was performed by using 2 mL of MeOH in disposable plastic tubes.

The solution was evaporated to dryness using a stream of nitrogen in a sample concentrator (Stuart, Cole-Parmer, USA). The residue was dissolved in 100 μL of H_2_O. An HPLC gradient liquid chromatograph (Shimadzu, Japan) with a QTRAP 6500+ mass detector (Sciex, Canada) was used for analysis of pesticides (see section 2.2.2).

### 3.4 Monitoring of DEET in Plants

Monitoring of DEET in plants was conducted twice a year at the same locations as surface water samples. The collection was carried out in plastic resealable bags. The samples were kept in a refrigerator at 4 °C until analysis. Subsequently, 25 g of the sample was weighed, and 100 mL of water was added to the sample. The sample was mixed in a blender and dried at 50 °C in an oven, and 1 g of dry matter was taken for subsequent analysis.

The dried plant sample was subsequently processed in the same way as for the sediment sample. In the first step, the QuEChERS method (see section 2.3.1) and in the second step a SPE extraction (see section 2.3.2) were performed. The sample was subsequently analyzed in the same way as in the case of a surface water sample (see section 2.2.2).

### 3.5 Monitoring of DEET in Gray Water

Gray water samples were collected once a month at the Hotel Zámek Vale with GPS coordinates 49.1475489N, 16.0360278E. The gray water from washing machines and SPA showers were both sampled into 1 L glass bottles from plastic retention tanks (50 L, one for each type of gray water). The samples were transported to the laboratory and stored in the refrigerator at a temperature of 4 °C until further preparation and analysis. On the day of the analysis, the samples were mixed and about 5 mL of each sample was filtered using syringe filters (ProFill Regenerated Cellulose, 0.45 μm, Thermo Fisher Scientific), 950 μL was transported into a vial alongside 20 μL of the internal standard solution (concentration 1 mg L^–1^). The prepared samples were analyzed via HPLC-MS/MS.

The same gradient liquid chromatograph and mass spectrometer as in section 3.2.2 were used for the analysis of gray waters. For the chromatographic separation, Kinetex 2.6 μm XB-C18 100 Å, 100 × 2.1 mm analytical column guarded with a C18 guard column (both Phenomenex, Torrance, CA, USA) was used along with the binary mobile phase, which consisted of ultrapure water (MP A) and mixture of acetonitrile and methanol (1:1; *v*/*v*) (MP B), both acidified with formic acid to make 0.1% solutions. The analysis was 15 min long (the length of the analysis was determined by another 37 personal care product (PCP) analytes, that were simultaneously analyzed with DEET in the same run), and the applied gradient was as follows: 0–1 min, 5% B; 1–6 min; 5–75% B; 6–9 min, 75–98% B; 9–10.5 min, 98% B; 10.5–11 min, 98–5% B, 11–15 min, 5% B; the flow rate was 0.3 mL min^–1^ and the injection was 10 μL. The mass spectrometric conditions were the same as in section 2.2.2, the retention times of both MRM transitions and LOQ value are listed in Table 3. The quantification was done using the IS method; a six-point calibration curve (0.1 μg L^–1^ – 200 μg L^–1^) spiked with IS solution was used for this purpose. For the quality control check of each batch run, two QC standards (on 10 and 100 μg L^–1^ levels) were measured. The analytical method was validated in terms of linearity, LOQ, repeatability and accuracy. The repeatability and accuracy were investigated on three concentration levels: low (5 μg L^–1^), middle (50 μg L^–1^) and high (200 μg L^–1^) and with the exception of the accuracy of the low point (121%), all of the deviations of accuracy were smaller than 15%; the RSDs (indicators of repeatability) were all smaller than 10%.

**Table 3:** Description of Collection Points

				water		sludge	
analyte	Q1 (*m*/*z*)	Q3 (*m*/*z*)	retention time (min)	LOD (μg L^–1^)	LOQ (μg L^–1^)	LOD (μg kg^–1^)	LOQ (μg kg^–1^)
DEET 1	192.185	118.600	6.78	0.01	0.03	26.17	82.19
DEET 2	192.106	90.800					

### 3.6 Monitoring of DEET in WWTP

#### 3.6.1 Monitoring of DEET in WWTP Inlet and Outlet Waters

Inlet and outlet water samples were collected in glass bottles monthly at the Ostrava WWTP (Czech Republic). The GPS coordinates are 49.8541128N, 18.2484061E. The samples were transported to the laboratory and stored in the refrigerator at the temperature of 4 °C until further preparation and analysis. The day of the analysis, the samples were mixed and about 5 mL of each sample was filtered using syringe filters (ProFill Regenerated Cellulose, 0.45 μm, Thermo Fisher Scientific), 950 μL were transported into vial alongside 20 μL of the inner standard solution (concentration 1 mg L^–1^). The prepared samples were analyzed in duplicates via HPLC-MS/MS using the same method as described in section 2.5.

#### 3.6.2 Monitoring of DEET in WWTP Sludge

Prior the analysis of DEET in sewage sludge samples, its extraction from the sludge matrix was done using accelerated solvent extraction. The hygienized sludges were sampled in PP containers (approximately 500 g) at the same WWTP as inlet and outlet WWTP waters in section 2.5.1, transported to the laboratory and stored in the refrigerator at the temperature of 4 °C until further preparation and analysis. Before the extraction, the sludge samples were thermally dried at 105 °C using the moisture analyzer (DLB 160–3A, KERN and SOHN GmbH, Balingen, Germany) and homogenized with batch mill (Tube Mill control, IKA, Staufen, Germany). The accelerated solvent extraction of sludge samples was done using Dionex ASE 300 Accelerated Solvent Extractor (Thermo Fisher Scientific, San Jose, CA, USA); the implied method was adopted from ref 23. Two g of dried sewage sludge sample was placed into the 10 mL extraction cell along with 1 g diatomaceous earth. The samples were extracted with 4 mL of a mixture of acetonitrile and ultrapure water (1/1 *v*/*v* with 0.1% formic acid) followed by extraction with 4 mL of acetonitrile, 2-propanol, and ultrapure water mixture (3/3/4 *v*/*v*/*v* with 0.1% formic acid). The extraction temperature was set to 50 °C, and the time of extraction was 10 min for each solvent-mixture. From the final combined extract (8 mL), 1 mL was put into a glass vial and analyzed via HPLC-MS/MS using the same method as described in section 2.5. The extraction efficiency of this procedure for DEET was 145% and all results were recalculated accordingly.

### 3.7 Ecotoxicity of DEET

#### 3.7.1 *Vibrio fischeri* Bioluminescence Inhibition

The bioluminescence inhibition test using liquid-dried luminescent bacteria *Vibrio fischeri* (Hach Lange GmbH, Germany) was carried out according to EN ISO 11348–3:2007 ^24^ standard. DEET solution was prepared by dissolving 1 g of DEET in 25 mL of methanol and supplemented with ultrapure water to a total volume of 1 L, and the salinity of the solution was adjusted by adding 20 g of NaCl per liter. Methanol had to be used due to the low solubility of DEET in water. Luminescence was measured in duplicate with a LUMIStox 300 luminometer (Hach Lange GmbH, Germany) at 15 °C using a LUMIStherm thermoblock (Hach Lange GmbH, Germany). A dilution series was prepared with eight steps increasing the dilution by a factor of 2 to obtain DEET concentrations ranging from 3.9 to 1000 mg L^–1^. The luminescence values were obtained after 15- and 30 min exposition. The EC50 value, representing the concentration of DEET causing 50% luminescence inhibition, was calculated using the dose–response function fit in Origin 2018b software.

#### 3.7.2 *Sinapis alba* Root Growth Inhibition

The root growth inhibition test using *Sinapis alba* seeds was performed with a DEET solution prepared with 1 g of DEET dissolved in 25 mL of methanol brought to a final volume of 1 L with ultrapure water. A series of 2-fold serial dilutions was made to obtain DEET concentrations ranging from 15.6 to 1000 mg L^–1^. The test was carried out in triplicate with 20 seeds and 5 mL of DEET solution for each repetition. To assess the effect of methanol in the solution, a treatment with only 2.5% methanol in water was also tested. The test was carried out at 20 °C (thermostatically controlled Lovibond ET 619–4/140Liter cabinet, Tintometer GmbH, Germany), in the dark and for 72 h. The average root length was then evaluated by image analysis using ImageJ software.^25^ By comparison with the mean root length in the blanks, root growth inhibition was calculated for all treatments and the EC50 concentration was determined by fitting the data with the dose–response function in Origin 2018b.

#### 3.7.3 *Eisenia andrei* Acute Toxicity Test

The acute toxicity test, specifically the paper contact toxicity test with earthworms *Eisenia andrei*, was carried out in accordance with OECD Test No. 207 (OECD, 1984). Therein, glass vials were lined with 8 × 8 cm square of filter paper moistened with 1 mL of DEET dissolved in ethanol, after evaporation rehydrated with 1 mL of deionized water and sealed with parafilm after inserting individual earthworms. A preliminary range-finding test was made with concentrations up to 50 wt % DEET, corresponding to 6.88 mg cm^–2^. However, two to 3 orders of magnitude lower concentrations were finally used to cover the entire range between zero and 100% mortality. The acute toxicity test consisted of five treatments with concentrations of DEET ranging from 0.039 to 0.625 wt % (0.005–0.078 mg cm^–2^) and a control treatment with pure ethanol, each performed in ten replicates involving one worm per vial. The test temperature of 20 °C was provided by a thermostatically controlled cabinet (Lovibond ET 619–4/140Liter, Tintometer GmbH, Germany). The test was conducted in the dark for 48 h, and then mortality of the worms was assessed. Median lethal concentration LC50 was computed by fitting mortality/concentration data with a dose–response function in Origin 2018b.

## 4 Results and Discussion

### 4.1 Monitoring of DEET in Surface Water

The concentrations of DEET at each sampling point in each month are shown on Figure 1 and in Table 4. The GPS coordinates of each sampling point are listed in Table 1. DEET monitoring was carried out over a period of 14 months, from September 2023 to October 2024, to determine the effect of the seasonal agricultural period. The maximum concentration of DEET found reached the value of 32.18 μg L^–1^.

**Table 4:** Concentration of DEET in Surface Water in Individual Locations and Months

	Bohumín - Odra (river)	Bohumín - Záblatí (pond)	Bohuslavice - Bohuslavický potok (stream)	Opava - Kateřinský potok (stream)	Ostrava - Opava (river)	Potštát - Harta (pond)	Potštát - Rybník u hřiště (pond)
month of sampling	*c* (μg L^–1^)	*c* (μg L^–1^)	*c* (μg L^–1^)	*c* (μg L^–1^)	*c* (μg L^–1^)	*c* (μg L^–1^)	*c* (μg L^–1^)
9/2023	<LOQ	0.005	0.81	2.56	0.41	<LOQ	<LOQ
10/2023	2.85	<LOQ	1.37	0.71	2.16	1.65	2.24
11/2023	0.70	1.03	1.92	0.21	0.67	0.60	0.58
12/2023	1.64	<LOQ	2.14	<LOQ	<LOQ	1.97	1.05
1/2024	<LOQ	<LOQ	0.85	<LOQ	<LOQ	<LOQ	<LOQ
2/2024	<LOQ	<LOQ	<LOQ	<LOQ	<LOQ	0.32	0.23
3/2024	19.50	6.23	2.55	4.92	1.60	1.05	1.33
4/2024	5.07	2.36	1.69	0.70	2.02	0.53	0.59
5/2024	<LOQ	0.11	0.19	<LOQ	0.19	0.14	0.11
6/2024	5.21	<LOQ	28.83	<LOQ	1.82	4.33	9.39
7/2024	3.50	1.23	4.79	0.36	5.39	1.20	0.71
8/2024	4.59	0.51	2.62	0.43	2.17	1.22	0.80
9/2024	<LOQ	<LOQ	<LOQ	<LOQ	32.18	<LOQ	<LOQ
10/2024	<LOQ	<LOQ	<LOQ	0.06	<LOQ	0.11	<LOQ

**Figure 1:**
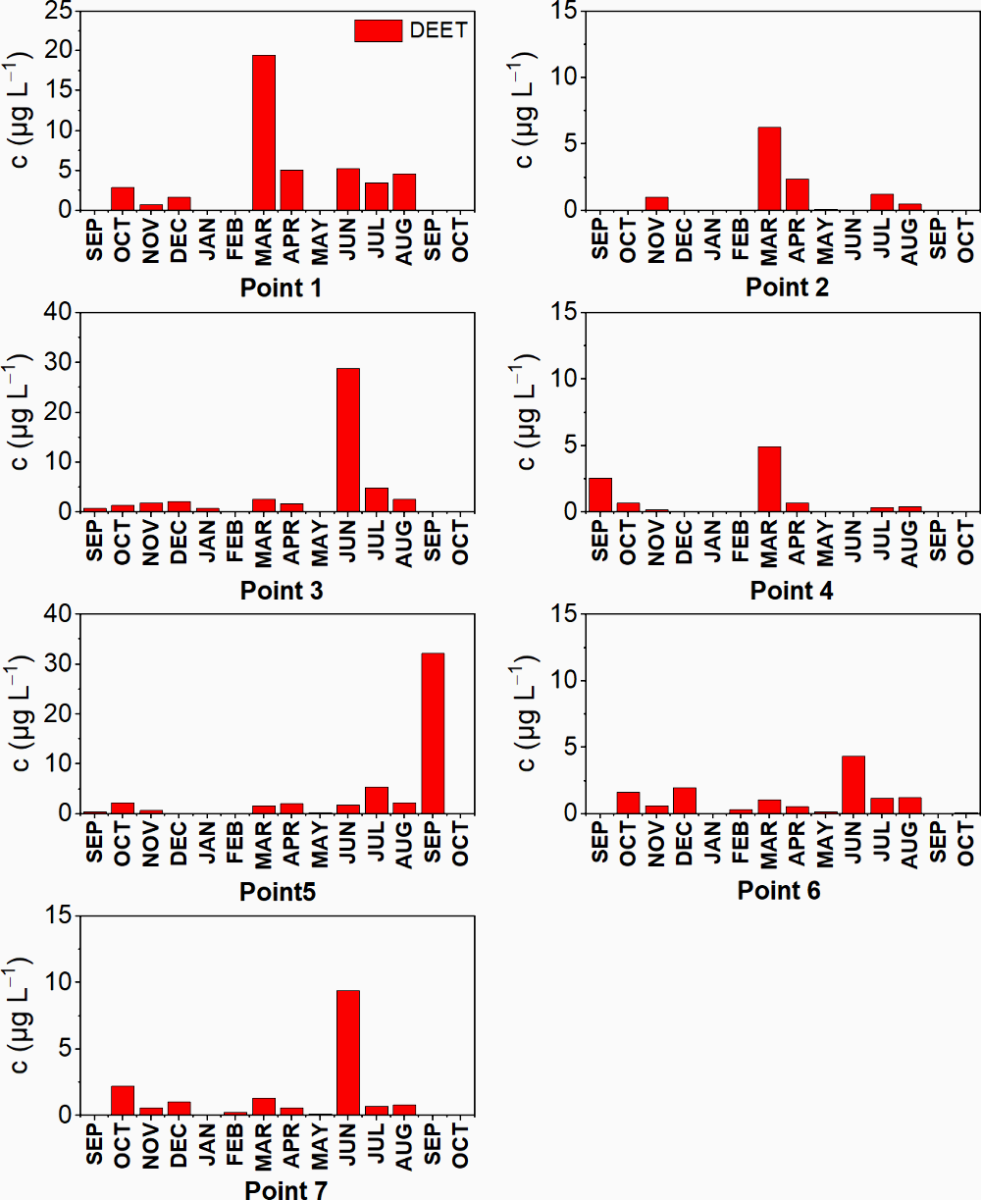
Concentration of DEET in surface water in individual locations and months.

The highest concentrations of DEET were measured especially in the summer months,^10^ which is associated with increased use of repellents and confirms the conclusions of other studies.^19,26^ Increased concentrations also occurred in March, when the number of fishermen and hunters around watercourses increases. In winter, pollution was minimal,^27^ except for December 2023, when DEET increased in four locations due to melting snow with subsequent runoff from surrounding fields.

The highest concentrations were detected at individual sampling points: Bohumín - Odra (river) 19.50 μg L^–1^ (March 2024); Bohumín - Záblatí (pond) 6.23 μg L^–1^ (March 2024); Bohuslavice - Bohuslavický potok (stream) 28.83 μg L^–1^ (June 2024); Opava - Kateřinský potok (stream) 2.56 μg L^–1^ (September 2023); Ostrava - Opava (stream) 5.39 μg L^–1^ (July 2024); Potštát - Harta (pond) 4.33 μg L^–1^ (June 2024); and Potštát - Rybník (pond) 9.39 μg L^–1^ (June 2024). Potštát - Harta (pond) and Pottát - Rybnk (pond) are located not far from each other; for that reason only small differences in DEET concentration at these sites can be seen in Table 4.

In September 2024, floods occurred in the Czech Republic, which caused dilution of substances in water; DEET was detected in only one place this month (Ostrava - Opava (stream)). In October, it was detected only in some places at low concentrations. Despite the fact that DEET was not detected in water in some places, it was detected in sediment and plants, see sections 3.2 and 3.3.

Numerous studies have monitored DEET in surface waters and found highly variable concentrations. For example, the highest reported levels are USA 3.7 μg L^–1^; Asia 24 μg L^–1^; Europe 1.29 μg L^–1^; and Oceania 0.49 μg L^–1^.^6^ Other findings include ranges: China <0.2–107 ng L^–1^; Singapore 1.4–527 ng L^–1^; South Korea 2–88 ng L^–1^; USA 1616.5 ng L^–1^; Japan 36 ng L^–1^.^28^ In Indonesia, high concentrations of DEET were measured in the Jakarta River (30–24000 ng L^–1^) and Jakarta Bay water (10–1100 ng L^–1^).^29^ In Lake Balaton in Hungary, the maximum was 1.57 μg L^–1^.^30^ These studies confirm that DEET concentrations vary considerably by location and season, as was also observed in our research. The seasonal variation of pesticides in surface water has also been observed for other pesticides.^31^

### 4.2 Monitoring of DEET in Sediment

In order to demonstrate the potential transport of DEET from surface water to other environmental compartments, this study was supplemented with the qualitative determination of DEET in sediment and plant samples. Sediment samples were taken 4 times a year at regular intervals. The sampling points are identical to the water sampling points listed in Table 1. Sampling was carried out in October 2023, February 2023, May 2024, September 2024, and October 2024. The detection of DEET in individual months in sediment is highlighted in Table 5.

**Table 5:** Concentration of DEET in Sediments and Plants in Individual Locations and Months

month of sampling	Bohumín - Odra (river)	Bohumín - Záblatí (pond)	Bohuslavice - Bohuslavický potok (stream)	Opava - Kateřinský potok (stream)	Ostrava - Opava (river)	Potštát - Harta (pond)	Potštát - Rybník u hřiště (pond)
Plants							
5/2024	**√**	**√**	**√**	**√**	**√**	**√**	**√**
6/2024	<LOD	<LOD	<LOD	<LOD	<LOD	<LOD	<LOD
9/2024	<LOD	<LOD	<LOD	<LOD	<LOD	<LOD	<LOD
10/2024	**√**	**√**	**√**	**√**	**√**	**√**	**√**
Sediment							
10/2023	**√**	<LOD	**√**	<LOD	**√**	**√**	**√**
2/2024	**√**	**√**	**√**	**√**	**√**	**√**	**√**
5/2024	**√**	<LOD	**√**	**√**	**√**	**√**	**√**
8/2024	**√**	**√**	**√**	**√**	**√**	**√**	**√**
10/2024	**√**	**√**	**√**	**√**	**√**	**√**	**√**

DEET was detected in October 2023 at almost all locations except Bohumn - Odra (river) and Ostrava - Opava (stream), probably due to the use of repellents by fishermen and higher precipitation. In February 2024, DEET appeared at all locations, probably due to melting snow and runoff from the surrounding area. In May, it was detected except for Bohumn - Záblatí (pond). In September and October 2024, DEET was again present at all locations, which is related to its medium stability and the possibility of accumulation in sediments.^2^

Several studies of DEET in sediments have confirmed that it is a ubiquitous compound, especially in locations popular for fishing and recreational activities, such as a study from Australia, which measured DEET concentrations in the range of 2.7–5.7 μg kg^–1^ dry matter in the Herbert and Daintree rivers.^32^ Similar results were obtained in a study from Texas, which demonstrated the presence of DEET in sediments of Corpus Christi Bay during both winter and summer months.^33^

While DEET photolysis can possibly occurs under sunlight in surface water^34^ this phenomenon does not happen in deeper layer of sediments where light does not penetrate. Based on its physicochemical properties, DEET is expected to be moderately mobile in the soil column and its half-life is measured in days to weeks.^12^ In soil, biodegradation by bacteria or fungi is a possible way for DEET elimination.^35^ However, despite the low accumulation of DEET in soils, there is a risk of DEET leaching into groundwater, which often serves as a source of drinking water.^36,37^

### 4.3 Monitoring of DEET in Plants

DEET monitoring in riparian plants was conducted in May, June, September, and October 2024 at the same locations as in water and sediment (Table 1), only qualitatively to determine the relationship between DEET in water, sediment, and plants. The detection of DEET in individual months in plants is highlighted in Table 5.

In May 2024, DEET was detected in all locations, which can be explained by plant growth in contaminated soil after the winter. In June 2024 and September 2024, when newly cut grass was sampled, DEET was not found in plants in September. In October 2024, DEET reappeared at all locations, probably due to flooding in September that removed vegetation and washed DEET-containing materials into the surrounding area. The detection of DEET in October may also represent only surface residues on the plants.

The ability of plants to absorb DEET has been demonstrated in crops such as tomatoes, corn and wheat, where bioaccumulation occurred to a steady state.^38^ Studies on the effects of DEET on wild plants are limited, although some report its absorption by roots and its presence in leaves or fruits (10–170 ng g^–1^ in hydroponics, 0.06–9.3 ng g^–1^ in the field).^39^ DEET can affect plant growth, for example by reducing protein and chlorophyll content, which can negatively affect photosynthesis.^40^ Since there is a real possibility of DEET being introduced into the soil in sewage sludge,^41^ it is necessary to look more deeply into its effect on plants, either alone or in combination with other micropollutants.

### 4.4 Monitoring of DEET in Gray Water

Monitoring of DEET in gray water took place from October 2023 to August 2024; the gray water was sampled in two locations: at the outlet of the SPA showers and the outlet of washing machines. The detected concentrations of DEET are highlighted in Figure 2 and summarized in Table 6.

**Table 6:** Concentration of DEET in Gray Water in Individual Locations and Months

	showers	the washing machine
month of sampling	*c* (μg L^–1^)	*c* (μg L^–1^)
10/2023	<LOQ	0.13
11/2023	<LOQ	0.22
12/2023	0.16	0.08
1/2024	0.07	0.05
2/2024	<LOQ	0.13
3/2024	0.21	0.07
4/2024	0.51	0.6
5/2024	0.18	0.16
6/2024	<LOQ	<LOQ
7/2024	<LOQ	<LOQ
8/2024	<LOQ	15.81

**Figure 2:**
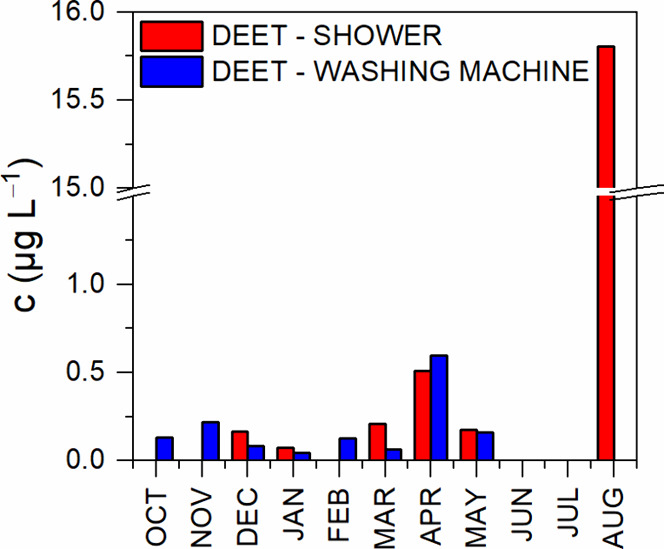
Concentration of DEET in gray water in individual locations and months.

Gray water is defined as wastewater from households, e.g., from showering, laundering clothes and kitchen sinks; gray water does not include wastewater from toilets.^42^ Gray water reuse is widely discussed as a suitable technology for drought mitigation, but the presence of micropollutants, such as DEET, prevents it from returning directly to the environment without any prior purification.^43^

In the gray water sampled from the SPA shower, DEET was detected and quantified in December 2023 and January, March, and May 2024. The concentrations were all below μg L^–1^, the highest concentration was measured in April of 2024, reaching 0.51 μg L^–1^. Strangely enough, the months in which the DEET was quantified were winter and spring months, which contradicts the expectation of DEET occurrence in gray and wastewaters mainly in summer months due to its highest consumption. Sampling graywaters in hotels and public facilities encompasses the variability in people, thus representing society-wide usage of DEET better than sampling just one household’s gray water. In the gray water sampled from the washing machine, DEET was detected every month, with the exception of June and July 2024. The highest concentration occurred in August of 2024, when the value had risen to 15.81 μg L^–1^, which supports the premise of summer months being the primal months of DEET consumption.

There is a general lack of data regarding the concentrations of DEET in gray water; only two papers have reported the concentrations according to our knowledge. Turner et al.^43^ measured DEET once a day for 1 week; the gray water combined the bathroom, kitchen, and laundry discharges and came from a single household. The concentrations oscillated between 1.2 and 1.8 μg L^–1^ (compared to our range of 0.07 to 0.51 μg L^–1^ for showers and 0.05 to 15.81 μg L^–1^ for laundry), but the concentrations are problematically comparable to our findings due to the discrepancies in the sampling periods (7 days vs several months). The other study by Muniz Sacco et al.^44^ reported a single concentration of 0.142 μg L^–1^ in a sample of real light gray water.

### 4.5 Monitoring of DEET in WWTP

Samples from the Ostrava WWTP were collected on a monthly basis from May 2024 to August 2024. The detected concentrations of DEET are illustrated in Figure 3 and summarized in Table 7. DEET was detected and quantified in every sample taken at the influent to the WWTP; the concentration ranged from 0.08 μg L^–1^ (June 2024) to 4.84 μg L^–1^ (August 2024). On the other hand, DEET was detected and quantified in the effluent only in August of 2024, in other months, the concentrations were below the LOD. The absence of detectable amounts of DEET in effluent waters in May, June and July of 2024 suggests that DEET is efficiently removed during the treatment processes, although a longer period of time is needed for removal of higher concentrations; this is supported by August influent and effluent concentrations, 4.84 μg L^–1^ and 1.73 μg L^–1^, respectively. August was also the only month when DEET was detected and quantified in the WWTP sludge with a concentration of 4.89 μg kg^–1^. The occurrence of DEET in WWTP in different countries differ. Liu et al.^45^ reported concentration of 9.568 μg L^–1^ in the influent waters of Beijing WWTP and 0.76 μg L^–1^ in the effluent. In this work, the measured concentrations of DEET in influent (I) and effluent (E) is compared to concentrations measured in WWTP in different countries, e.g., Greece (I: 0.084–1.038 μg L^–1^; E: 0.005–0.240 μg L^–1^),^46^ Vietnam (I: 0.500–1.200 μg L^–1^; E: 0.300–0.400 μg L^–1^),^47^ United States of America (I: 0.050–0.200 μg L^–1^; E: 0.03–0.200 μg L^–1^)^10^ and Spain (I: not detected–0.683 μg L^–1^; E: not detected–0.492 μg L^–1^).^48^ Such high concentrations as those measured in Beijing can be attributed to very high population, but the occurrence of DEET and other PPCPs is not always positively related to population density. The removal efficiencies (RE) of DEET in different WWTPs are highly inconsistent, some works report RE as high as 90%^49^ and some very poor (27.4%).^50^ Liu et al.^45^ reported almost 100% RE, stating that >50% of removal of DEET can be attributed to sorption on particles during secondary sedimentation processes.

**Table 7:** Concentration of DEET in the WWTP at the Inflow, Outflow, and in the Sludge in Individual Months

	inlet	outlet	sludge
month of sampling	*c* (μg L^–1^)	*c* (μg L^–1^)	*c* (μg kg^–1^)
5/2024	0.33	<LOQ	<LOQ
6/2024	0.08	<LOQ	<LOQ
7/2024	2.73	<LOQ	<LOQ
8/2024	4.84	1.73	4.89

**Figure 3:**
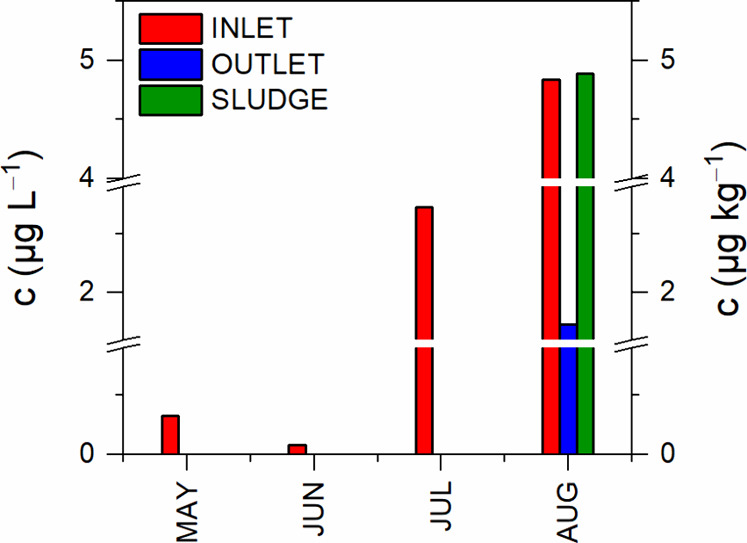
Concentration of DEET in the WWTP at the inflow, outflow, and in the sludge.

### 4.6 Ecotoxicity

#### 4.6.1 *Vibrio fischeri* Bioluminescence Inhibition

For luminescent marine bacteria *V. fischeri*, the EC_50_ of DEET was determined to be 0.155 ± 0.007 g L^–1^ and 0.106 ± 0.004 g L^–1^ after 15 min and 30 min exposure, respectively (Figure 4). The small amount of methanol present in the solution is assumed not to have any inhibitory effect, as methanol is known to be toxic to bacteria cells only at concentrations of several volume percent.^51^ In other studies, the EC_50_ was determined to be 67.9 mg L^–1^ ^51^ and 21.2 mg L^–1^ ^52^ for DEET. However, the latter value is not reliable due to the fact that the maximum test concentration was 10 mg L^–1^, and the EC_50_ was obtained by extrapolation. It is important to note that EC_50_ values are derived from experiments conducted on living organisms and exhibit a certain degree of variability. However, there is a clear agreement in terms of order of magnitude with the results reported by other authors. On a broader scale, the acute toxicity of DEET toward other aquatic species is described in the EC range of 4 to 388 mg L^–1^ and the chronic no-observed effect concentrations (NOEC) range from 0.5 to 24 mg L^–1^,^12^ which is several orders of magnitude higher than the concentrations found in surface waters in this study.

**Figure 4:**
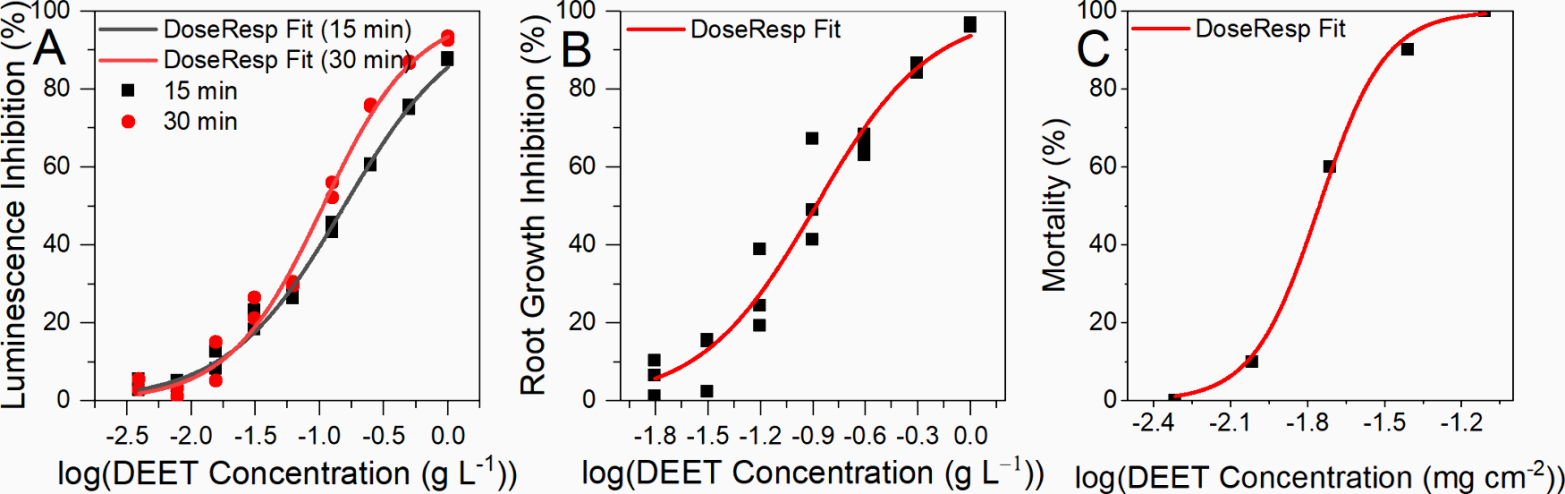
(A) *V. fischeri* bioluminescence inhibition after 15 min and 30 min exposure fitted with a dose–response function. (B) *S. alba* root growth inhibition fitted with a dose–response function. (C) *E. andrei* acute toxicity fitted with a dose–response function.

#### 4.6.2 *Sinapis alba* Root Growth Inhibition

Based on the root growth inhibition test with *S. alba*, the EC_50_ of DEET was determined to be 0.130 ± 0.009 g L^–1^, which is similar to the results obtained for *V. fischeri* (Figure 4). It was confirmed using blank samples that methanol at a concentration of 2.5% had no inhibitory effect on root growth. To the authors’ knowledge, this is the first study of DEET ecotoxicity in relation to *S. alba*. A rare study by Xi and Zacharia^18^ examining the effect of DEET on radish (*Raphanus sativus*) confirms an effect on seed germination at a concentration of 0.01% (which corresponds to 0.1 g L^–1^), with the presence of DEET reducing and also delaying the germination. These values indicate that the effect of DEET on plants is more likely to occur when DEET-containing products are used in nature (sparse point sources of pollution) than when indirect exposure via wastewater, in which the DEET content is much lower.

#### 4.6.3 *Eisenia andrei* Acute Toxicity Test

In the acute toxicity test on *E. andrei*, the LC_50_ of DEET was calculated as 0.0175 ± 0.0005 mg cm^–2^, which corresponds to a concentration of 1.121 g L^–1^. No mortality was observed at DEET concentrations below 10 μg cm ^–2^, but tissue damage and impaired motility were observed in the earthworms. Increasing mortality and morphological changes, including body constriction, fragmentation, and visible bleeding, were observed at concentrations ranging from 10 to 78 μg cm ^–2^ (Figure 4). Similar damage was observed after exposure of earthworms *Eudrilus eugeniae* to the pesticides chlorpyrifos, cypermethrin, triazophos, and deltamethrin with LC_50_ values of 0.165, 0.066, 0.076, and 0.031 μg cm ^–2^, respectively, Singh et. al and Tiwari at. al.^53,54^ These LC_50_ values are 2 orders of magnitude lower than those determined in the present study, indicating much lower toxicity of DEET intended for skin application compared to pesticides usually applied in agricultural fields. Although sensitivity varies between earthworm species, and *E. fetida*, together with *E. andrei*, are among the less-sensitive species,^55^ the difference in EC_50_ at the 2 orders of magnitude cannot be due to differences in sensitivity alone.

To the authors’ knowledge, the toxicity of DEET on earthworms has not yet been published, probably because this repellent is not intended for application to terrestrial environments. Studies with other species of worms are more recent and mainly focus on roundworms *Caenorhabditis elegans*.^56,57^ However, these are not classic ecotoxicity studies but rather an elucidation of the mechanism by which DEET acts on *C. elegans* at the genetic level.

Although DEET does not pose a primary threat to the terrestrial environment, particularly due to the mode of application, it should be kept in mind that it may be introduced into the soil through the application of sewage sludge. It would be worthwhile to determine DEET concentrations in such treated soils and relate them to terrestrial toxicity results.

## 5 Conclusions

Based on the monitoring of DEET in surface waters, sediment, and plants on the banks, its transport in the environment has been proven. In the case of months in which there is increased application, its concentration in water is many times higher. Based on the higher stability of DEET, it is also possible to detect it in sediment and therefore in plants that grow from it.

Application to clothing and human skin is considered to be the primary source of DEET in graywater and wastewater and subsequently surface water and sediment. This is because DEET enters these environments through water from showering or bathing after application and laundering of clothes.

The results of ecotoxicity tests have indicated the potential for a detrimental impact of DEET on aquatic and soil environment. Although acute toxicity has been observed at concentrations several orders of magnitude higher than those found in the environment, long-term ecotoxicological effects at much lower concentrations and, in particular, synergistic effects cannot be excluded. Furthermore, elevated concentrations of DEET were observed in the vicinity of the WWTP outfall.

## Data Availability

Data will be made available on request.
